# Impact of new traumatic or stressful life events on pre-existing PTSD in traumatized refugees: results of a longitudinal study

**DOI:** 10.3402/ejpt.v7.32106

**Published:** 2016-11-09

**Authors:** Katrin Schock, Maria Böttche, Rita Rosner, Mechthild Wenk-Ansohn, Christine Knaevelsrud

**Affiliations:** 1 Center Ueberleben, Berlin, Germany; 2 Department of Clinical Psychology and Psychotherapy, Freie Universität Berlin, Germany; 3 Department of Clinical and Biological Psychology, Katholische Universität Eichstätt-Ingolstadt, Germany

**Keywords:** Traumatized refugees, trauma, stressful life event, trauma-associated stimuli, posttraumatic stress disorder

## Abstract

**Background:**

A significant proportion of trauma survivors experience an additional critical life event in the aftermath. These renewed experiences of traumatic and stressful life events may lead to an increase in trauma-related mental health symptoms.

**Method:**

In a longitudinal study, the effects of renewed experiences of a trauma or stressful life event were examined. For this purpose, refugees seeking asylum in Germany were assessed for posttraumatic stress symptoms (PTS), Posttraumatic Stress Diagnostic Scale (PDS), anxiety, and depression (Hopkins Symptom Checklist [HSCL-25]) before treatment start as well as after 6 and 12 months during treatment (*N*=46). Stressful life events and traumatic events were recorded monthly. If a new event happened, PDS and HSCL were additionally assessed directly afterwards. Mann–Whitney *U*-tests were performed to calculate the differences between the group that experienced an additional critical event (stressful vs. trauma) during treatment (*n*=23) and the group that did not (*n*=23), as well as differences within the critical event group between the stressful life event group (*n*=13) and the trauma group (*n*=10).

**Results:**

Refugees improved significantly during the 12-month period of our study, but remained severely distressed. In a comparison of refugees with a new stressful life event or trauma, significant increases in PTS, anxiety, and depressive symptoms were found directly after the experience, compared to the group without a renewed event during the 12 months of treatment. With regard to the different critical life events (stressful vs. trauma), no significant differences were found regarding overall PTS, anxiety, and depression symptoms. Only avoidance symptoms increased significantly in the group experiencing a stressful life event.

**Conclusion:**

Although all clinicians should be aware of possible PTS symptom reactivation, especially those working with refugees and asylum seekers, who often experience new critical life events, should understand symptom fluctuation and address it in treatment.

**Highlights of the article:**

- Within a longitudinal design, refugees currently in treatment are compared concerning symptom course after new traumatic or stressful events.
- New stressful life events and traumatic events increase posttraumatic, anxious, and depressive symptoms in the first 6 months after the events.
- Stressful and traumatic events overall did not show differential symptom exacerbations.
- Only avoidance symptoms were an exception; stressful life events were associated with an increase in PTS avoidance symptoms.
- Because reducing avoidance is the pivotal mechanism of psychotherapy with PTSD patients, clinicians should anticipate PTS symptom reactivation when patients are re-exposed to significant stressful stimuli, especially with asylum seekers and refugees, who often experience new stressful events.

Posttraumatic stress disorder (PTSD) is one of the most common mental disorders in the aftermath of a traumatic life event. As defined by the diagnostic criteria of the DSM-5 (American Psychiatric Association [APA], 2013), a traumatic event is an exposure to actual or threatened death, serious injury, or sexual violation; it (1) is experienced directly, (2) is experienced by witness in person, (3) has occurred to a close family member/friend (with the actual or threatened death being either violent or accidental), or (4) is experienced firsthand through repeated or extreme exposure to aversive details (not including media, pictures, television, or movies, unless work related).

Studies show that a substantial proportion of the general population experience multiple traumatic events during their lifespan (Carper et al., 2015; Ogle, Rubin, Berntsen, & Siegler, 2013; Spitzer et al., 2008). High-risk populations such as war-affected people, refugees, firefighters, or police officers often experience traumatization sequentially (Harvey et al., 2015; Levy-Gigi, Richter-Levin, Okon-Singer, Keri, & Bonanno, 2015), which has a significant impact on the development of mental disorders, for example, PTSD, depression, and anxiety (Kartal & Kiropoulos, 2016; Pinto, Henriques, Jongenelen, Carvalho, & Maia, 2015; Stanley, Hom, & Joiner, 2016). Several studies have provided evidence that individuals suffering from PTSD in the aftermath of an initial trauma are vulnerable to increased PTSD symptoms after subsequently experiencing a new traumatic event (Bramsen, Van Der Ploeg, & Boers, 2006; Fossion et al., 2015; Hantman & Solomon, 2007; Kinzie, Boehnlein, Riley, & Sparr, 2002).

Kinzie et al. (2002) examined the impact of a new traumatic event on traumatized refugees with and without pre-existing PTSD. The authors showed that 2 months after the additional trauma, an increase in posttraumatic stress symptoms (PTS) and comorbid disorders was observed in all participants. Participants already suffering from PTSD showed the highest increase in PTSD symptoms. After 5 months, symptoms reduced to the baseline level (i.e., before the additional trauma happened). A prospective epidemiological study underpinned the impact of a new trauma on pre-existing PTSD (Breslau, Borges, Hagar, Tancredi, & Gilman, 2009), reporting that individuals who already developed PTSD showed a significantly higher increase in symptoms after a new subsequent trauma. The authors conclude that the presence of PTSD after an initial trauma has a greater impact on PTSD symptoms following exposure to a subsequent trauma compared with the characteristics of this subsequent trauma.

This increase in PTSD symptoms in the aftermath of a subsequent traumatic event is not well defined at present. Researchers define this increase as either reactivation/reactualization (Bramsen et al., 2006; Fossion et al., 2015; Heir & Weisaeth, 2006; Kinzie et al., 2002) or retraumatization (Maercker & Mehr, 2006; Orth & Maercker, 2004). These concepts can be distinguished regarding the length and quality of PTSD symptoms. Reactualization/reactivation is described as a less severe or moderate form of PTS after the experience of a new traumatic event, leading to a short-term symptom increase with which the victim is able to cope by herself/himself (Maercker & Rosner, 2006). Retraumatization is described as a significant exacerbation of PTS (Maercker & Michael, 2009; Orth & Maercker, 2004), based upon three core elements: first, the individual has to develop a PTSD in the aftermath of the initial trauma; second, after experiencing an additional trauma, the intrusion symptoms refer to and are related to the initial trauma rather than the subsequent trauma (Hantman & Solomon, 2007); and third, a retraumatization is associated with a significantly longer-lasting increase of the pre-existing PTSD symptoms compared with a reactivation/reactualization (Schock, Rosner, & Knaevelsrud, 2015). However, this definition is not well established and empirical evidence for the three core elements is missing.

Research has focused on the impact of sequential traumatization on mental health in high-risk populations. Nonetheless, these populations are also confronted with a high level of stressful life events in the aftermath of their initial traumatic event (e.g., Harvey et al., 2015; Levy-Gigi et al., 2015). As Turner (2015) stated, the problems that refugees and asylum seekers face do not end with their arrival in a safe country. This holds strong relevance because especially refugees and asylum seekers—with an estimated PTSD rate of 30.6% (Steel et al., 2009)—are facing such multiple stressful life events in their country of exile (i.e., post-migration stressors). To date, in this highly afflicted group, very little is known about the impact of stressful life events that do not meet the DSM-5 trauma criteria but are often associated with the initial index trauma (e.g., “asylum interviews” and fear of repatriation). The so-called asylum interview is a substantive interview about a person’s reasons for claiming asylum in Germany conducted by the German immigration authorities. In this interview, refugees have a “duty to cooperate”; they are required to disclose all facts related to their asylum application, including the history of their persecution and their reasons for fleeing their home country. In a study on the effects of the asylum interview within the asylum process, Schock et al. (2015) found a significant increase in PTSD intrusion symptoms as well as a significant decrease in PTSD avoidance and hyperarousal symptoms. In addition, post-migration stressors like uncertainty regarding the legal status (Nickerson, Steel, Bryant, Brooks, & Silove, 2011; Ryan, Dooley, & Benson, 2008) and fear of repatriation and persecution (Herlihy, Scragg, & Turner, 2002; Steel, Frommer, & Silove, 2004) resulted in increased PTSD symptoms. Furthermore, integration problems were closely associated with psychological symptoms in (traumatized) asylum seekers (Schick et al., 2016). These results underpin the findings of an earlier meta-analysis (Porter & Haslam, 2005) examining poorer mental health outcomes for refugees owing to post-displacement conditions (e.g., institutional accommodation, and repatriation).

Summarizing the above, it remains unclear whether the subsequent event that causes an increase in pre-existing PTSD symptoms (i.e., retraumatization or reactivation) needs to be related exclusively to a traumatic experience or whether a stressful life event could also elicit the same psychopathological reaction.

Thus, this study investigates the PTS symptom course after experiencing a subsequent critical life event (traumatic or stressful). The study has two aims: (1) to compare the PTS, depression, and anxiety symptom course in a group of patients with PTSD who either experienced a critical life event or did not experience such event and (2) to evaluate the impact of either a traumatic or stressful life event on the PTS symptom course with the aim of differentiating between symptom patterns. Based upon prior research, we hypothesize that individuals experiencing either new traumatic or stressful events show an increase of pre-existing PTS symptoms compared to individuals without both events. Furthermore, we hypothesize that individuals with pre-existing PTS who experience a new trauma show a higher PTS symptom increase than those who experience a stressful life event.

## Methods

### Sample

This study was conducted at the Berlin Center for Torture Victims, Germany, from November 2009 to June 2011. All participants were patients of the outpatient clinic and received long-term multimodal treatment including psychotherapy (i.e., psychoeducational, eclectic trauma-focused, and resource-oriented interventions) as well as medical interventions and support by social workers related to their history of war-associated trauma and/or torture in their home. As an integral part of their treatment, patients received standardized assessments of their psychopathology at the beginning of the treatment (t1), as well as after 6 (t2) and 12 (t3) months during treatment. In addition, patients were asked to complete an additional questionnaire if a subsequent trauma or stressful event happened. This study is based on anonymized data, collected as part of routine medical and psychotherapeutic care. Specific categories of personal data (health data) were collected, saved, and used for the purpose of treatment in the context of the provided psychotherapeutic support. This also includes the evaluation of the treatment outcomes. Thus, for the evaluation and anonymous publication, no special permission (informed consent) is required because it is covered by the purpose of the data collection (for treatment).

Overall, 94 patients were included in the longitudinal study, of whom 23 had experienced either a subsequent trauma according to the DSM-IV-TR definition (APA, 2000) or a subsequent stressful (trauma-associated) event between November 2008 and June 2010 (i.e., life event group—LE group). Each LE group participant was matched with a participant from the non-LE group (*n*=71), that is, a participant who had not experienced a new trauma or stressful (trauma-associated) event during the period. Matching criteria were sex, age, years of education, asylum status, and number of traumatic events.

## Measures

All measures used were translated and subsequently back-translated by interpreters blinded to the original version. Discrepancies were discussed and adjusted. Therefore, all questionnaires were available in all study languages, which were Chechen, English, Farsi, French, Kurdish, Russian and, Turkish. They were administered using multilingual computer-assisted self-interview (Knaevelsrud & Mueller, 2008), a computer-based touchscreen diagnostic program that allows participants to read each item and the range of possible answers in their native language and/or—in case of illiteracy—listen to each item being read aloud. In addition, all diagnostic sessions were conducted with the assistance of professional interpreters (e.g., to explain the study and respond to queries). Participants completed all following questionnaires at baseline and every 6 months during treatment (i.e., three assessment points). Participants with a new experienced trauma or stressful event during these 12 months also completed Posttraumatic Stress Diagnostic Scale (PDS) and Hopkins Symptom Checklist (HSCL) after reporting such an event. Only the “List of Life Events” was assessed every month to identify potential stressful or traumatizing events.

### Traumatic events

Participants identified the respective traumatic event from a trauma list comprising items derived from the PDS Part I (Foa, 1995) and the Harvard Trauma Questionnaire event list (Mollica et al., 1992). In sum, the list contains 23 different types of traumatic events. The participants indicated whether they had experienced, witnessed, or heard about any of the specified traumatic events. A total number of different self-experienced or witnessed traumatic events was calculated by creating dichotomous variables (i.e., 1=experienced or witnessed and 0=not exposed to trauma).

### Life events

The “List of Life Events” contained well-known post-migration stressors (Silove, Steel, McGorry, & Mohan, 1998) as well as potential stressful and traumatizing events, which were generated based upon clinical expert interviews regarding potential stressful and traumatic situations for refugees. On a monthly basis, participants were asked about the occurrence of a stressful or traumatizing event (0=no and 1=yes). In sum, eight events and one open category were included in this list especially developed for this study. They were rejection of asylum application and impending deportation, interview with the Federal Office for Migration and Refugees, war in the home country, physical abuse, serious illness (patient himself/herself), death or serious illness of relatives (in home country), verbal threats, and confrontation with the place where the trauma happened (travel to the home country). If participants experienced at least one of these events, PDS and HSCL-25 were subsequently assessed in addition to the three regular assessment points.

### Posttraumatic stress disorder

The Posttraumatic Diagnostic Scale (Foa, 1995) assesses PTSD symptom severity as well as the intrusion, avoidance, and hyperarousal clusters of DSM-IV PTSD (APA, 1994). The self-report questionnaire comprises 17 items assessing the frequency of PTSD symptoms during the last 4 weeks on a four-point Likert scale ranging from 0=*never* to 3=*nearly always*. The index for symptoms severity ranges (i.e., sum scores) from 0 to 51 were (1–10 mild, 11–20 moderate, 21–35 moderate to severe, and 36–51 severe PTSD). Sum scores were also calculated regarding the symptom clusters, (intrusion: range 0–15; avoidance: range 0–21, and hyperarousal: range 0–15). The scale has very good internal consistency (alpha=0.92) and test–retest reliability (*r*=0.74) and was validated across several populations, for example, refugees (e.g., Schnyder et al., 2015; Heeren et al., 2012). The PDS can be repeatedly administered to monitor symptom changes and is recommended as a particularly useful tool for screening and assessing PTSD (Foa, Cashman, Jaycox, & Perry, 1997).

### Anxiety and depression

Symptoms of anxiety and depression were assessed with the HSCL-25 (Derogatis, Lipman, Rickels, Uhlenhuth, & Covi, 1974; Khuon & Lavelle, 1987), which comprises a 10-item subscale for anxiety symptoms and a 15-item subscale for depression symptoms. The respondents were asked to indicate the extent to which they had been bothered by each symptom in the previous week on a four-point Likert scale from 1=*not at all* to 4=*extremely*. Both severity scores were calculated by forming means of all corresponding items with a cut-off score of 1.75, indicating clinical significant symptom severity. The 25-item scale was adapted for use in refugee populations and has very good validity and reliability.

## Data analysis

Statistical analyses were performed using the Statistical Package for the Social Sciences, version 22.0 (SPSS 22.0) for Macintosh. Given the small sample size, we estimated missing values using the EM algorithm for maximum likelihood estimation with incomplete information (Graham, 2009). The data were reported as means and standard deviations.

In a first step, study participants were assigned to either the group that experienced an additional critical life event within the three regular assessment points (LE group) or the group that experienced no additional critical life event during this period (non-LE group). Because the data did not follow a normal distribution, we ran Mann–Whitney *U*-tests for the three assessment points to examine the difference between the groups with (LE group) and without (non-LE group) a new traumatic/stressful event, taking into account the experience of a new traumatizing event as a fixed factor. Symptom changes between the assessment points t1 (baseline), t2 (after 6 months of treatment), and t3 (after 12 months of treatment) were analyzed with Wilcoxon tests for dependent variables.

In a second step, to analyze the impact of a new trauma versus a new stressful life event, we divided the LE group (*n*=23) into two subgroups according to the DSM-5 definition of trauma (APA, 2013). We assigned the following traumatizing experiences to the subgroup “traumatic event” (*n*=10): pictures and videos of massacres in their home country with friends or family members being involved (*n*=4); family members being abducted in their home country (*n*=3); death of a close friend in the country of exile (*n*=1); and experience of physical abuse in the country of exile (*n=*2). Consequently, we assigned the following stressful experiences to the subgroup “stressful life event” (*n=*13): failure of asylum application (*n*=6); return to the home country and the places where the trauma had happened (*n*=2); new political unrest/war in the home country; fears for the safety of family members (*n*=4); and lawsuit in the country of exile (*n*=1). In this study, none of the participants reported more than one additional traumatic and stressful event, respectively. All additional traumatic or stressful events occurred between the second (t2) and third (t3) assessment point, aside from two stressful life events (failure of asylum application) and one traumatic event (death of a close friend in the country of exile). Because of the small number of events occurring between t1 and t2, the influence of time point was not statistically examined. When comparing the influence of a traumatic or stressful event, the time periods (pre and post) in which the event happened (from t1 to t2 and from t2 to t3) were pooled as pre and post. The chance of finding a statistically significant difference between the groups is small owing to the study’s small sample size. Therefore, we also report effect sizes for non-significant effects with a significance level of *p*<0.10 because of type II error (i.e., failing to detect an effect that is present). To examine the difference between the groups with a new traumatic and new stressful event, we also ran Mann–Whitney *U*-tests. *P*-values reported for the group differences statistics are Bonferroni corrected and one-tailed.

## Results

### Sociodemographic characteristics and psychopathology

As the Mann–Whitney *U*-tests indicated, the non-LE group and LE group did not significantly differ in any sociodemographic characteristics (Table 1). The gender ratio was almost balanced in both groups with 43% (*n*=10) women in the non-LE group and 52% (*n*=12) women in the LE group. In both groups, 61% (*n=*14) of the participants were married. Most participants came from Iran (non-LE group: *n*=9, 39%; LE group: *n*=6, 26%), followed by the Balkan region (non-LE group: *n*=4, 17%; LE group: *n*=6, 26%) and Turkey (non-LE group: *n*=3, 13%; LE group: *n*=4, 18%). Because of their small numbers, participants from other countries of origin were combined into a single category (non-LE group: *n*=7, 31%; LE group: *n*=7, 31%). None of the participants had a secure residence status and all were currently unemployed.

**Table 1:** Sociodemographic characteristics of the non-life event (*n=*71) and life event (*n=*23) groups

	Non-LE group		LE group		
			
	Mean	SD	Mean	SD	Group comparison (*U*, *p*)
Age (in years)	36.35	10.84	35.83	12.2	252.00, 0.55
Education (in years)	8.4	2.1	7.7	2.2	241.00, 0.88
Number of children	1.7	1.5	1.9	1.4	236.50, 0.53
Months since arrival in Germany	12.4	2.8	11.3	1.7	218.00, 0.30
No. of traumatic events	17	5.6	18	4.3	275.00, 0.78

LE, life event; non-LE, non-life event; SD, standard deviation.

At the baseline, participants (*n*=94) reported on average 17 traumatic events (range 5–22), with 11 based upon direct experience (range 3–22), that is, the trauma happened to the participant personally. The most frequently reported directly experienced traumatic events were being close to death (83.4%, *n=*38), torture (76.2%, *n*=35), and war (66.7%, *n*=30).

All participants (*n*=97) were diagnosed with PTSD. The average PDS score at baseline (t1) was *M=*38.9 (Standard deviation, SD=8.6) in the non-LE group and *M*=39.4 (SD=9.2) in the LE group, indicating a high PTSD symptom severity. Similarly, both groups reported severe anxiety and depression symptoms above a cut-off score of 1.75 (non-LE group: *M*=39.4, SD=9.2; LE group: *M*=39.4, SD=9.2).

## Longitudinal outcome of non-LE and LE groups

Regarding the non-LE group, the data revealed a significant reduction for all psychopathological variables from the baseline to the 6-month assessment, with large effect sizes from *r*=0.56–0.88: intrusion *T*(23)=2.03, *p*<0.05; avoidance *T*(23)=3.04, *p*<0.005; hyperarousal *T*(23)=2.32, *p*<0.05; anxiety *T*(23)=2.78, *p*<0.05; and depression *T*(23)=3.29, *p*<0.005. The same significant decrease was found for the LE group: intrusion *T*(23)=2.17, *p*<0.05; avoidance *T*(23)=4.00, *p*<0.001; hyperarousal *T*(23)=3.96, *p*<0.001; anxiety *T*(23)=2.79, *p*<0.05; and depression *T*(23)=3.52, *p*<0.001 (Table 2).

**Table 2:** Means (M) and standard deviation (SD) for outcome measures at baseline, 6- and 12-month assessments

		Baseline (t1) M (SD)	6 months (t2) M (SD)	12 months (t3) M (SD)
PTSD sum	Non-LE group LE group	38.9 (8.6)^a^ 39.4 (9.2)^a^	26.4 (9.4)^b^ŧ 27.4 (9.3)^b^ŧ	23.7 (8.5)^b^ 24.2 (8.2)^b^
Intrusion	Non-LE group	10.91 (3.2)^a^	9.4 (2.5)^b^	8.8 (2.2)^b^
	LE group	11.5 (3.5)^a^	8.8 (3.0)^b^	9.9 (3.2)^b^
Avoidance	Non-LE group	15.2 (4.8)^a^	8.7 (4.2)^b^Ŧ	7.7 (4.6)^b^
	LE group	15.8 (4.1)^a^	10.9 (4.4)^b^Ŧ	9.0 (4.3)^b^
Hyperarousal	Non-LE group	11.13 (3.5)^a^	8.6 (3.2)^b^	8.2 (3.5)^b^
	LE group	11.6 (2.9)^a^	7.7 (2.9)^b^	7.3 (2.5)^b^
Anxiety	Non-LE group	2.8 (0.59)^a^	2.2 (0.60)^b^	1.9 (0.64)^c^
	LE group	2.8 (0.49)^a^	2.2 (0.72)^b^	2.2 (0.68)^b^
Depression	Non-LE group	2.7 (0.65)^a^	2.1 (0.71)^b^	1.9 (0.65)^b^
	LE group	2.8 (0.66)^a^	2.1 (0.81)^b^	1.9 (0.54)^b^

a,b,c Means within a row that share a superscript do not differ at *p*<0.05 (one-tailed)

Ŧ, ŧ Significant group differences between LE and non-LE groups at *p*<0.05 (one-tailed).

From the 6-month to the 12-month assessment, only anxiety significantly decreased in the non-LE group (*T*(23)=3.17, *p*<0.05). All other psychopathological variables showed no significant symptom changes in both groups.

### Comparison of non-LE and LE groups

There were no differences in any psychopathological variables between the non-LE and LE group at the baseline assessment (t1, Table 2). Calculated effect sizes concerning the difference between the groups were all very small (*r*=0.01– *r*=0.14). At t2, overall PTSD (*U*=194.00, *p<0*.05, *r=0*.32) as well as avoidance (*U*=185.00, *p*<0.05, *r*=0.88) significantly differed between the two groups, indicating that the LE group shows a higher symptom level compared to the non-LE group.

As the sample size is small, we report also on trends. The LE group showed a trend to a higher symptom level of intrusion (*U*=201.00, *p*<0.07, *r*=0.29), avoidance (*U*=206.00, *p*<0.09, *r*=0.27), and overall PTSD (*U*=207.50, *p*<0.06, *r*=0.26) in the LE group compared to the non-LE group, although no significant group differences are shown for t3. This shows that the experience of a new critical event has a negative impact on the symptom course of PTSD.

Anxiety and depressive symptoms did not differ at baseline and 6-month assessments. However, a significant difference and a large effect size were found for anxiety symptoms at the 12-month assessment (t3; *U*=164.50, *p*<0.02, *r*=0.46), indicating a higher anxiety level for the LE group compared to the non-LE group, but not for depressive symptoms.

### Impact of trauma or stressful life event

Trauma and stressful event groups do not differ in any PTSD cluster at the regular assessment point before the subsequent life event happened (pre) and the effect sizes are also very small (*r*=0.00–0.03) (Table 3).

**Table 3:** Means (standard deviations) of outcome variables for the trauma and stressful life event group during the occurrence of a subsequent trauma/stressful event

		Pre (t1 or t2)	Trauma or stressor (TS)	Post (t2 or t3)
Intrusion	Trauma group	9.0 (3.7)^a^	10.9 (2.4)^b^	9.0 (2.4)^a^
	Stressor group	8.5 (4.1)^a^	12.5 (2.6)^b^	9.9 (4.2)^c^
Avoidance	Trauma group	10.2 (4.6)^a^	12.8 (3.2)^b^	6.9 (3.0)^c^
	Stressor group	9.8 (5.7)^a^	15.4 (3.7)^b^	9.1 (4.2)^a^
Hyperarousal	Trauma group	8.7 (2.8)^a^	9.3 (4.1)^b^	6.6 (1.2)^c^
	Stressor group	9.4 (4.1)^a^	11.7 (2.8)^b^	7.0 (2.6)^c^
PTSD sum	Trauma group	27.9 (9.6)^a^	33.0 (7.8)^b^	22.5 (5.5)^c^
	Stressor group	27.6 (12.4)^a^	39.6 (6.8)^b^	26.8 (9.2)^a^
Anxiety	Trauma group	2.4 (0.67)^a^	2.7 (0.57)^a^	1.7 (0.30)^b^
	Stressor group	2.3 (0.81)^a^	3.0 (0.41)^b^	2.0 (0.53)^a^
Depression	Trauma group	2.4 (0.56)^a^	2.6 (0.64)^a^	1.9 (0.45)^b^
	Stressor group	2.1 (0.85)^a^	2.8 (0.51)^b^	1.9 (0.51)^a^

Pre=regular assessment point during the treatment (i.e., either t1 or t2), TS=assessment point directly after the subsequent trauma or stressful event, post=regular assessment point during treatment (i.e., either t2 or t3).

a,b,c Means within a row that do not share a superscript differ at *p*<0.05 (*t*-test, one-tailed).

In the stressful event group, the analyses revealed significantly higher overall PTS symptoms directly after the experience of the critical event in comparison to the trauma group (*U*=30.00, *p*<0.03, *r*=0.45). Furthermore, a trend towards a significant difference was found for the avoidance (*U*=34.00, *p=*0.05, *r*=0.40), indicating that patients experiencing a stressful life event showed higher avoidance symptoms directly after the life event compared to the trauma group. Effect sizes of this trend were moderate to large. Especially, the items “trying not to think about, talk about, or have feelings about the event” were endorsed (*U*=23.00, *p*<0.005, *r*=0.60). Furthermore, moderate—albeit non-significant—effect sizes were found for the differences between both groups for intrusion, (*U*=39.00, *p=*0.10, *r*=0.34).

At the regular assessment point after the occurrence of the subsequent life event (post), data analyses showed no significant difference between the two groups in any psychopathological measurement.

## Time period of symptom increase

All significant group differences (avoidance and overall PTSD) observed directly after the subsequent trauma or stressful event disappeared within 6 months. No significant group differences occurred for the regular assessment after the subsequent trauma/stressful event (Table 4).

**Table 4:** Group differences (U) with effect sizes (r) of outcome variables for the trauma and stressful life event group during the occurrence of a subsequent trauma/stressful event

	Pre *U* (*p*, *r*)	Trauma or stressor *U* (*p*, *r*)	Post *U* (*p*, *r*)
Intrusion	62.00 (0.85, −0.03)	39.00 (0.10, −0.34)	50.00 (0.38, −0.19)
Avoidance	62.00 (0.85, −0.03)	34.00 (0.05*, −0.40)	44.50 (0.20, −0.27)
Hyperarousal	65.50 (0.97, −0.00)	42.00 (0.15, −0.30)	55.00 (0.53, −0.13)
PTSD sum	63.00 (0.90, −0.02)	30.00 (0.03*, −0.45)	41.50 (0.14, −0.30)
Anxiety	57.50 (0.64, −0.09)	55.50 (0.55, −0.12)	38.50 (0.15, −0.29)
Depression	49.50 (0.34, −0.20)	51.50 (0.40, −0.16)	62.50 (0.88, −0.03)

*U*=Mann−Whitney test; p=level of significance

* =p≤0.05.

## Discussion

This study explored the PTS symptom course after experiencing a subsequent traumatic or stressful life event. Two questions were investigated: (1) Do PTSD patients with and without a subsequent critical life event differ in PTS, anxiety, and depressive symptom courses? and (2) In what way does the experience of a subsequent trauma or stressful life event influence the symptom course of PTS, anxiety, and depression symptoms?

### Comparison of PTSD patients with and without a subsequent critical life event

Significant group differences were found after 6 months in the PDS total score. This can be attributed to the significant group differences in the avoidance in the LE group. A possible explanation for this symptom increase in the LE group can be derived from the fear network model (Foa & Kozak, 1986; Foa & Rothbaum, 1998), which enables the rapid detection of threat as well as the immediate initiation of a defensive reaction and underlies the neuronal processing of fear. It is assumed that activation of the fear network by triggering stimuli—for example, reminders of the original trauma like a subsequent trauma or stressful life event—activates the network. Avoidance symptoms result from trying to attempt to suppress such activation (i.e., symptoms of re-experiencing). In addition, the avoidance of genuinely threatening stimuli or situations is a key characteristic of adaptive fear (Krypotos, Effting, Kindt, & Beckers, 2015). This means that PTSD patients will try to avoid intrusive memories, as well as actively avoiding any confrontation with trauma-associated stimuli that may cause intrusive memories (Brewin & Holmes, 2003; Williams & Moulds, 2007). In both groups, significant improvement in mental health was observed over a 6-month period (t1 to t2). No significant symptom improvements were found from the 6 to 12-month assessment, whereas solely anxiety symptoms in the non-LE group showed a significant decrease from t2 to t3. Significant group differences were found at the 6-month assessment in the total score of PTS symptoms, which can be ascribed to the significant group differences in the PTS avoidance symptoms. However, at the 12-month assessment, no significant group differences in the total score of PTS symptoms were found. Nonetheless, participants remained highly symptomatic at this assessment point. This decrease of symptoms may be explained by several reasons. First, all participants received psychotherapeutic treatment, that is, the increase of symptoms after a subsequent critical event could have been treated adequately. Second, Kinzie et al. (2002) showed that following a significant increase in PTSD symptoms in refugees after the 9/11 terror attacks, the symptoms had returned to their baseline clinical status within 5 months, thus indicating a natural decrease of symptoms over time following a subsequent trauma. Yet, significant group differences were found in the anxiety symptoms at t3, indicating that the LE group showed significantly more anxiety symptoms at t3. Possibly participants who experienced a new critical event felt more vulnerable and insecure owing to the subsequent experience of unstable circumstances, which may lead to increased anxiety symptoms. This assumption could be underpinned by research showing preliminary results suggesting that anxiety and the sense of invulnerability are negatively associated (Kleiman et al., 2015).

### Differential impact of a subsequent trauma versus a stressful life event

Contrary to our expectations, new stressful life events influenced the symptom course more than the experience of a new traumatic event. Significant group differences in avoidance and the PTSD total score directly after the experience of the trauma or stressful life event emerged, indicating that new stressful life events have a greater impact on the PTSD symptoms than a new trauma. This goes in line with previous research showing that study participants reporting an event classified as non-traumatic indicated significantly greater levels of PTSD symptoms than those who experienced a traumatic event (Criterion A; DSM-IV-TR) (Cameron, Palm, & Follette, 2010; Gold, Marx, Soler-Baillo, & Sloan, 2005). Boals and Schuettler (2009) took into account both the A1 (experiencing a traumatic event) and A2 criteria (response to traumatic event involving intensive fear, hopelessness, or horror), comparing the PTSD scores of those who met both criteria (A1 and A2) with those who only fulfill criterion A1 (i.e., experience of a traumatic event without a response involving intensive fear, hopelessness, or horror). They showed that there was very little to no relation between the experience of a trauma (A1) and PTSD symptoms when A2 criterion was taken into consideration. The authors conclude that it may not be the nature of the event itself (A1) that leads to higher psychological distress, but rather the emotional or psychological reaction to the event. In our study, most of the mentioned stressors were related to an unsecure residence status and thus insecurity concerning their life and their future. The high emotional distress associated with this insecurity might potentially resemble similar distress as captured with the initial A2 criterion in DSM-IV.

In addition, previous studies indicate that a lack of social acknowledgment of the traumatic experience predicts the development of PTSD (Brewin, Andrews, & Valentine, 2000; Mueller, Moergeli, & Maercker, 2008; Wagner, Keller, Knaevelsrud, & Maercker, 2012). The experience that one’s account and suffering is invalidated by rejecting the asylum claim may result in feelings of powerlessness, humiliation, helplessness, and fear (Simms, Grös, Watson, & O’Hara, 2008). In this regard, research indicates that a rejection of the residence is associated with high levels of PTSD, anxiety, and depression (Mueller, Schmidt, Staeheli, & Maier, 2011).

Another possible explanation for the significant impact of stressful life events is the concept of event centrality, which refers to an individual’s interpretation of how central a negative event is to his/her life story and narrative identity (Rubin, Boals, & Hoyle, 2014). Event centrality is seen as one of the strongest factors associated with PTSD (Boals, Hayslip, Knowles, & Banks, 2012; Brown, Antonius, Kramer, Root, & Hirst, 2010; Rubin et al., 2014). In a study with a variety of stressful life events, Boals and Ruggero (2015) suggested that initial levels of event centrality affect subsequent changes in PTSD symptoms (Boals & Ruggero, 2015). In terms of our study, it can be assumed that the stressful life events such as the failure of an asylum application or new political unrest/war in the home country are strongly associated with the individual’s identity, affecting the living circumstances in the near future in terms of uncertainty, invalidation of their experiences, and separation from family members. However, we did not assess this construct explicitly and thus we can only speculate about its potential influence.

The significantly higher PTSD total score in the stressful life event group directly after the experience of a stressful event can be attributed to the significantly higher avoidance in this group. The avoidance cluster of PTSD relates to a specific avoidance of reminders or cues about the traumatic event. In our study, especially the items “trying not to think about, talk about, or have feelings about the event” were endorsed by the participants experiencing a stressful event. At the regular assessment—that is, the data collection every 6 months—the results showed no significant group differences in PTSD sum score or any symptom cluster, nor in anxiety and depressive symptoms. The decrease in avoidance symptoms from the assessment directly after the stressful life event until the regular assessment point may be ascribed to therapeutic interventions during that time, focusing on reducing avoidance behavior (Ehlers & Clark, 2008). However, it could also be assumed that the avoidance symptoms might also naturally decrease within 6 months, as Kinzie et al. (2002) showed. However, because of the lack of an untreated control group we could not draw any conclusions about the course of the avoidance symptoms without psychological treatment.

### Limitations

The main limitation of this study is the small sample size, which increases the risk of type II errors. Thus, further research based upon larger samples is required to clarify whether differences in symptoms change according to the nature of different events or stimuli. Furthermore, the assessment of the traumatic or stressful event itself was based upon the patient’s self-report or the therapist’s recognition, whereby we risk not capturing all events or stimuli. However, with the monthly reminder for the therapist and a standardized list with potential traumatic and stressful events, we tried to optimize the operationalization. Another limitation is that we only focus on the occurrence of a new traumatic or stressful event. It is an important aspect for future studies to rate the impact of each stressor. Differences in the impact of either a traumatic or stressful event could be another explanation why the life events group responded differently than the trauma group. Another limitation is that all participants were in treatment and the symptom increase after the traumatic or stressful life event was a topic in treatment. From a therapeutic perspective, reducing avoidance is the pivotal mechanism of psychotherapy with PTSD patients (Follette, Iverson, & Ford, 2009). In this context, Droždek (2015) recommends applying the use of multimodal and trauma-focused interventions sequentially in a course of the treatment trajectory. However, we cannot draw conclusions regarding whether the reactions and duration of the symptom increase would be the same in a sample without therapeutic support.

## Conclusion

Our findings contribute to the current literature on PTSD symptom course and refugees’ mental health in several respects.

First, our study results enhance the previous understanding of retraumatization by showing that stressful life events may also lead to a significant increase in pre-existing PTSD symptoms and not (only) an additional trauma according to criterion A (APA, 2013). Second, the results show that the experience with an additional stressful life event may lead to a significant increase in PTSD avoidance.

In addition, our study results underline the significant impact of additional critical life events. Because the experience of new traumatic events has been the most robust predictor of a chronic course of PTSD in a longitudinal study on traumatized adolescents (Perkonigg et al., 2005), it is essential that clinicians anticipate PTSD symptom reactivation when patients are re-exposed to significant stressful stimuli. This notion holds strong relevance in the therapeutic process, especially in therapeutic work with asylum seekers and refugees who are often exposed to new stressful events.
